# Pilot scale production, extraction and purification of a thermostable phycocyanin from *Synechocystis* sp. PCC 6803

**DOI:** 10.1016/j.biortech.2021.126459

**Published:** 2022-02

**Authors:** Anton Puzorjov, Suleyman Mert Unal, Martin A. Wear, Alistair J. McCormick

**Affiliations:** a SynthSys & Institute of Molecular Plant Sciences, School of Biological Sciences, University of Edinburgh, Edinburgh EH9 3BF, UK; b The Edinburgh Protein Purification Facility, University of Edinburgh, Edinburgh EH9 3JR, UK

**Keywords:** Ammonium sulfate, Chitosan, High pressure homogenisation, Scale-up, *Thermosynechococcus elongatus* BP-1

## Abstract

Phycocyanin (PC) is a soluble blue pigment-protein primarily harvested from the cyanobacterium *Arthrospira platensi*s. PC is in high demand from several industries, but its narrow stability range limits potential applications. Here, a pilot scale (120 L total) batch production, extraction and purification process for thermostable PC (Te-PC) from a *Synechocystis* sp. PCC 6803 'Olive' strain expressing the PC operon *cpcBACD* from *Thermosynechococcus elongatus* BP-1 on a self-replicating vector is presented. Batch cultivation without antibiotics had no impact on growth or Te-PC production and optimisation of growth conditions resulted in Te-PC contents of 75.3 ± 1.7 mg g DW^−1^. Wet biomass was harvested following chitosan-based flocculation with a 97 ± 2% efficiency, and Te-PC was extracted by high pressure homogenisation. Subsequent purification by heat-treatment and two-step ammonium sulfate precipitation removed chlorophyll and allophycocyanin contamination, resulting in Te-PC purities of 2.9 ± 0.7 and a mean Te-PC recovery of 84 ± 12%.

## Highlights

• *Synechocystis* sp. PCC 6803 produced thermostable phycocyanin from a self-replicating vector.
• Pilot scale (120 L) cultivation without antibiotics did not affect phycocyanin production.
• A biomass flocculation efficiency of 97 ± 2% was achieved using chitosan.
• Heat treatment during ammonium sulfate precipitation improved phycocyanin purity and recovery.

## Graphical abstract

**Figure:**
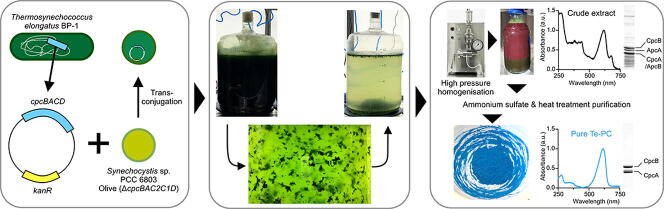

## 1 Introduction

Phycocyanin (PC) is a soluble, pigment-protein found within the light-harvesting phycobilisome complex of cyanobacteria and red algae. Purified PC is characterised by a bright blue colour and red fluorescence, and is primarily harvested from the filamentous cyanobacterium *Arthrospira platensis* (Spirulina). At present, PC is the only commercially available natural blue colourant, and is thus in high demand in the food and cosmetics industries, while its fluorescent and anti-oxidant properties are of interest to the biopharmaceutical, nutraceutical, and therapeutical sectors (Pagels et al., 2019).

A key factor limiting the broader commercial uptake of PC is the narrow pH and temperature stability range of PC from *A. platensis* (Puzorjov & McCormick, 2020). PC extracted from *A. platensis* denatures at temperatures exceeding 45 °C and/or outside a pH range of 4–7, which results in a loss of its blue colour, fluorescence and anti-oxidant properties (Pan-utai et al., 2018). In comparison, PC extracted from thermophilic organisms that grow optimally at temperatures at or above 45 °C has significantly improved thermostability properties (Puzorjov et al., 2021). For example, PC from the thermophilic cyanobacterial species *Synechococcus lividus* PCC 6715, *Thermosynechococcus elongates* BP-1 and *Synechococcus lividus* SyI is stable at 60 °C, 70 °C and 80 °C, respectively (Puzorjov et al., 2021), while PC extracted from the red alga *Galdieria phlegrea* retains its fluorescent properties up to 85 °C (Ferraro et al., 2020). However, the maximum growth rates of thermophiles and PC content per cell dry weight are relatively low (Liang et al., 2018), and establishing a standardised process to produce and extract high-quality PC from thermophilic species is challenging (Lang et al., 2020). In particular, cultivation of extremophiles at the higher temperatures needed for optimal growth requires a substantial energy investment (Puzorjov et al., 2021).

Previous work has demonstrated that the mesophilic model cyanobacterium *Synechocystis* sp. PCC 6803 (hereafter *Synechocystis*) can be engineered to host an autonomous self-replicating expression vector (e.g. RSF1010-based) to produce thermostable PC from *T. elongates* BP-1 (Te-PC) expressed at a growth temperature of 30 °C (Puzorjov et al., 2021, Vasudevan et al., 2019). The yields of Te-PC in the *Synechocystis* mutant strains were comparable to that of native PC in wild-type *Synechocystis*, while the thermostability properties matched those from *T. elongatus*. Recent work has demonstrated that the level of eYFP expression from an RSF1010-based vector was 3-fold higher than eYFP expression from chromosomal integrations by natural transformation (Nagy et al., 2021). Importantly, that study also showed that the self-replicating vector was retained for 42 days without antibiotic selection at lab scale, thereby suggesting that scale-up batch cultivation without antibiotic selection is feasible.

*Synechocystis* is considered a relatively fast-growing cyanobacterium with a maximum reported growth rate of 0.16 h^−1^ (4.3 h doubling time) and can achieve cell densities of 10.5 g DW L^-1^ under optimised photobioreactor, growth media and light conditions (van Alphen et al., 2018). Numerous lab-based studies have demonstrated the potential of *Synechocystis* as a green biofactory for small chemical production, such as terpenes and carbohydrates (Veerabadhran et al., 2021). Recently, peptides from plants, animals and prokaryotes have been expressed at 10–20% of total cellular protein, thus setting the foundation for *Synechocystis* as recombinant protein production platform (Zhang et al., 2021). However, to date scale-up production (10–1300 L) has only been demonstrated for the production of polyhydroxybutyrate (PHB) and hydrogen (Lindblad et al., 2019). To further promote *Synechocystis* as robust and scalable biofactory, cell harvesting and disruption methods must be demonstrated at larger scales.

The present work describes a pilot scale (120 L, consisting of six 20 L carboys) batch production, harvesting and purification process for Te-PC expressed in *Synechocystis* from a self-replicating RSF1010-based vector. The scalable downstream processing includes efficient biomass flocculation with the natural flocculant chitosan, optimised cell disruption using high pressure homogenisation and a novel purification protocol that combines heat-treatment and ammonium sulfate precipitation to take advantage of the thermostable properties of Te-PC.

## 2 Materials and methods

### 2.1 Culture strain

A previously generated *Synechocystis* sp. PCC 6803 'Olive' mutant (Δ*cpcBAC2C1D*) was transconjugated with a self-replicating (RSF1010-based) vector pPMQAK1-T carrying an expression cassette for the TeBACD operon from *T. elongatus* (CT.353 TeBACD, see supplementary materials) (Puzorjov et al., 2021). The expression cassette was assembled using the CyanoGate MoClo toolkit and consisted of the TeBACD operon driven by the native *Synechocystis* PC promoter (P_cpc560;_ part pC0.005) and terminator (T_cpc_, part pC0.078) (Puzorjov et al., 2021, Vasudevan et al., 2019). Cultures were screened for the presence of the CT.353 TeBACD by PCR as described in Gale et al. (2019). The oligonucleotides used for amplification are described in the supplementary materials.

### 2.2 Lab scale growth conditions and pilot scale batch culture

All experiments were performed in secure labs or rooms with appropriate biosafety risk assessments in place. Lab scale liquid cultures (<1 L) of the TeBACD transconjugant were maintained in sterile 1xBG11 medium supplemented with 50 μg mL^−1^ kanamycin under continuous white LED light (50 μmol photons m^2^ s^−1^) and 30 °C in an Infors Multitron-Pro (Infors HT) incubator shaken at 100 rpm and aerated with filter sterilised, water saturated atmospheric air (Puzorjov et al., 2021). For each batch culture experiment, fresh seed cultures (50 mL) were initiated in 100 mL conical flasks. At OD_750_ = 4.0 the cultures were transferred into 1 L conical flasks and 450 mL of 1x BG11 medium was added. The 500 mL cultures were again grown to OD_750_ = 4.0 and transferred into 20 L carboys containing 19.5 L of non-sterile 1x or 2xBG11 medium for pilot scale batch culture. In the first experiment, the carboys from the kanamycin group (kan) were supplemented with 50 μg mL^−1^ kanamycin. Liquid cultures in carboys were grown under continuous white LED light (150 or 300 μmol photons m^2^ s^−1^, 40 W, 4200 K) or progressive red LED light (684 nm with 24 nm full width at half maximum). The temperature inside the carboys was maintained at 30 ± 1 °C using heat pads (25 W). Cultures in the carboys were aerated with filter sterilised, water saturated atmospheric air supplemented with 2% CO_2_ at a flow rate of 3 L min^−1^ using a gas mixing system (GMS 150, Photon Systems Instruments). Each growth condition experiment was performed with three carboys (i.e. three independent biological replicates). OD_750_ was measured daily using a Biowave II Spectrophotometer (Biochrom WPA). To measure dry biomass, a total of 50 mL of culture was collected from each carboy every two days and quantified as described in Zavrel et al. (2018). The reduction in white light penetration at OD_750_ = 0.1 and OD_750_ = 1.0 was calculated as in Lea-Smith et al. (2014) by comparing white light (500 μmol photons m^2^ s^−1^) levels at 30 mm depth.

### 2.3 Phycobiliprotein concentration, purity and chlorophyll content

Te-PC and APC were extracted from the dry biomass and quantified using absorbance spectroscopy in 96-well flat-bottom (Chimney Well) μCLEAR plates on a FLUOstar OMEGA microplate reader (BMG Labtech) as described in (Zavrel et al., 2018). Total soluble protein content and chlorophyll *a* (Chl) content in the crude extract were estimated based on the absorbance at A_280_ and A_680_, respectively (Zavrel et al., 2018). Te-PC purity was calculated as the ratio of the Te-PC peak (A_615_) to the total soluble protein content (A_280_). Chlorophyll content was measured as described in Puzorjov et al. (2021).

### 2.4 Biomass flocculation

A stock solution of medium molecular weight chitosan (190–310 kDa, Sigma-Aldrich) was prepared by dissolving 10 g of chitosan in 1 L of 0.5% (v/v) acetic acid solution under continuous stirring for 24 h at room temperature. At the end of the growth experiment, the culture was adjusted to pH 6.8 using a weak solution of hydrochloric acid or by bubbling the culture with 10% CO_2_ at a flow rate of 1.2 L min^−1^ for 1 h. Chitosan solution was then added to each carboy (50 mg L^-1^ final concentration), which were shaken manually for 15 s. After 3 min the carboys were shaken for a further 15 s and left in the dark for 16 h at room temperature. After incubation, the OD_750_ of the supernatant without flocs was measured and the efficiency of the flocculation calculated. The supernatant was then removed using a plastic syphon pump without disturbing the sedimented cyanobacterial flocs. The remaining concentrated culture (1.5 L per carboy) was centrifuged at 5,000 *g* for 10 min and the supernatant was removed. The pellet was transferred into a pre-cooled glass blender (BL82AD40 PerfectMix+, Tefal) and resuspended in an equal volume of ice-cold pH adjusted phosphate-buffered saline (PBS, pH 8.8) for 3 min to wash off residual chitosan. The cells were then centrifuged and the pellet resuspended in PBS (pH 7.8) to reach the dry biomass concentration of 12.4–13.9 g DW L^-1^.

### 2.5 High pressure homogenisation

Cyanobacterial biomass was lysed following 1–3 passes through an Emulsiflex-C3 high pressure homogeniser (Avestin) at 10,000–25,000 psi. Lysed biomass was centrifuged at 17,600 *g* for 20 min at 4 °C to separate the crude extract from cell debris.

### 2.6 Ammonium sulfate precipitation

The crude extract was subjected to a two-step ammonium sulfate (AS, 99% pure, ACROS Organics) precipitation (Mogany et al., 2019). During the optimisation of the first precipitation step, AS was added to 100 mL of the crude extract to reach 15% of the AS saturation at 4 °C according to the online tool AS Calculator (https://www.encorbio.com/protocols/AM-SO4.htm) (EnCor Biotechnology Inc.) and stirred for 1 h. Samples (1.8 mL) were then taken in technical triplicates and centrifuged at 17,600 *g* for 30 min at 4 °C. The supernatant was carefully removed and the pellet was resuspended in an equal volume of PBS. Te-PC, total protein content and Chl contamination was quantified using a microplate reader as described in section 2.3. The AS concentration of the crude extract at 15% AS was then increased in 5% or 10% steps by adding additional AS. After each step (20, 25, 30, 40 and 50% AS saturation), the crude extract was stirred for 1 h before triplicate samples were collected and quantified as above. For the large-scale AS precipitation, AS was added to 450 mL of crude extract to reach 15% AS saturation at 4 °C and stirred for 1 h. The sample was then heat-treated as described in section 2.8, and centrifuged at 17,600 *g* for 30 min at 4 °C. The second step AS precipitation was performed by adding AS to the supernatant to reach 50% AS saturation at 4 °C and stirred overnight. The solution was then centrifuged at 17,600 *g* for 30 min at 4 °C and the pellet, containing Te-PC, was resuspended in a 20 mL of PBS and quantified as described above. The resuspended pellet was then dialysed (Biodesign D106 Cellulose Dialysis Tubing, MWCO 8 kDa) against 100x volume of ultra-pure water at 4 °C for 72 h. Dialysis buffer was refreshed after 24 and 48 h. Dialysed pure Te-PC extract was then freeze-dried over two days.

### 2.7 Size-exclusion chromatography and SDS-PAGE

Freeze-dried Te-PC powder was resuspended in 15 mM citric acid-trisodium citrate buffered to pH 6.0. The solution (250 µL, equivalent to 425 µg Te-PC) was injected onto a Superdex-200 10/300 GL size exclusion column (Cytiva), pre-equlibrated in 100 mM trisodium citrate, pH 6.0 at 8 ˚C, and run at 0.5 mL min^−1^. Absorption at 280 nm and 615 nm (2.5 sec integration) was measured using an ÄKTA™ pure 25 M2 liquid chromatography system (Cytiva) with UNICORN™ 7.3 software (GE, USA). Fractions of 0.5 mL were collected after 0.2 column volumes of delay post injection through the elution. A sub-aliquot of the initial resuspended Te-PC sample was diluted to A_615_ = 5.0 and subjected to sodium dodecyl sulfate polyacrylamide gel electrophoresis (SDS-PAGE) on a NuPAGE 12% (w/v) Bis-Tris (Invitrogen) protein gel and stained with 1% (v/v) Coomassie Blue in acetic acid/methanol. An equivalent amount of the crude PC extract was analysed for comparison.

### 2.8 Heat treatment purification of thermostable phycocyanin

For the heat treatment test experiment, the supernatant containing Te-PC extracts following the first step 15% AS precipitation and no-AS treated samples were incubated at 60 °C for 15 min on a heating block. Immediately after incubation, the samples were cooled down on ice. All samples were centrifuged at 17,600 *g* for 30 min at 4 °C and the supernatant was quantified using a microplate reader as described in section 2.3. After the heat treatment, AS was added to the no-AS samples to reach 15% of the AS saturation at 4 °C and stirred for 1 h. The samples were then centrifuged and the supernatant was quantified as described above. For large-scale application of heat treatment, 450 mL of the crude extract containing 15% AS (section 2.6) was incubated at 60 °C for 15 min in a water bath with continuous stirring. The sample temperature was monitored using an immersion glass thermometer. Immediately after incubation, the sample was cooled down on ice.

### 2.9 Thermal and pH stability of phycocyanin

Freeze-dried Te-PC powder or commercially available powdered PC samples extracted from *A. platensis* (LinaBlue, DIC; ScotBio Blue, ScotBio) were resuspended in 15 mM citric acid-trisodium citrate buffered to pH 3, 4, 5, 6 or 7 and diluted to 0.1 g L^-1^. Samples were incubated on a heating block at 40 °C or 70 °C. PC content (g L^-1^) was measured after 15, 30 and 60 min of incubation in percentage relative to the untreated control. Te-PC samples were also measured after 180 min of incubation. The fluorescence emission spectra of Te-PC (excited at 590 nm) were measured in black 96-well flat-bottom μCLEAR plates (Greiner Bio-One) on a CLARIOstar microplate reader (BMG Labtech).

## 3 Results and discussions

### 3.1 Biomass and thermotolerant phycocyanin productivity of the *Synechocystis* strain TeBACD

**Fig. 1:**
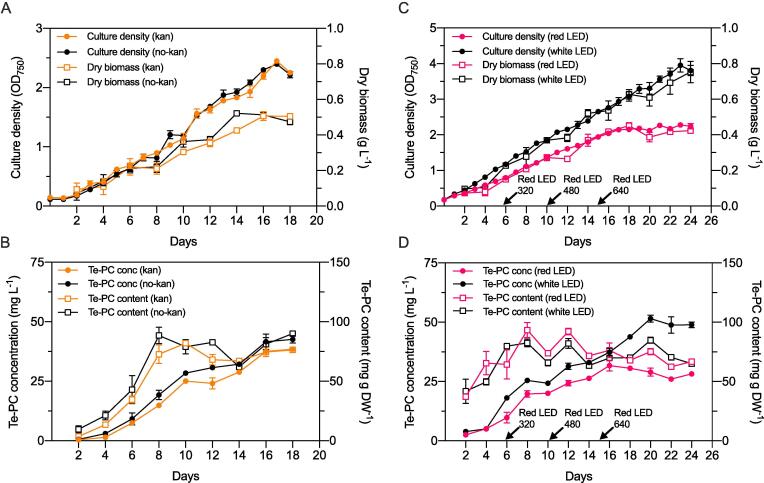
Growth analysis and phycocyanin content of the *Synechocystis* 'Olive' mutant transconjugant TeBACD. (A) The culture density (OD_750_) and dry biomass concentration (g L^-1^), and (B) Te-PC content (mg g DW^−1^) and Te-PC concentration (mg L^-1^) grown in white light (150 μmol photons m^−2^ s^−1^) and measured over 18 days in the presence (kan) or absence (no-kan) of kanamycin (50 µg mL^−1^) in BG11 medium. The mean ± SE PC content between days 8 and 18 was 73.4 ± 2.3 and 80.6 ± 4.1 mg g DW^−1^ for kan and no-kan cultures, respectively. (C) The culture density (OD_750_) and dry biomass concentration (g L^-1^), and (D) Te-PC content (mg g DW^−1^) and Te-PC concentration (mg L^-1^) grown in white light (300 μmol photons m^−2^ s^−1^) or progressive red light (red LED) and was measured over 24 days in 2xBG11 medium without kanamycin. The mean ± SE PC content between days 8 and 24 was 72.7 ± 2.8 and 75.5 ± 3.6 mg g DW^−1^ for cultures grown in white and red light, respectively. The intensity of red light at day 0 was 160 μmol photons m^−2^ s^−1^, which was then increased at day 6, 10 and 15 as shown along the x-axis. The error bars in culture density show the mean ± SE of three biological replicates. The error bars in dry biomass, Te-PC content and Te-PC concentration show the mean ± SE of three biological replicates. (For interpretation of the references to colour in this figure legend, the reader is referred to the web version of this article.)

Two pilot scale growth experiments were performed using a low-cost photobioreactor (PBR) design to examine the performance of the TeBACD strain and yields of Te-PC under different conditions. In the first experiment, TeBACD cultures were grown in 1xBG11 medium under white light (150 μmol photons m^−2^ s^−1^) in the presence or absence of kanamycin to assess the impact of the antibiotic on cyanobacterial growth and Te-PC production. During an 18-day cultivation period, no significant differences in culture density (OD_750_) or biomass accumulation (g DW L^-1^) were observed (*p* < 0.05, paired Student’s *t*-test), indicating that the presence of kanamycin had no negative effect on growth (Fig. 1**A**). Maximum culture densities of OD_750_ = 2.4 ± 0.1 were reached at 17 days, after which a decrease was seen in all cultures, likely due to nutrient depletion and/or light limitation (van Alphen et al., 2018).

The concentration of Te-PC (mg L ^-1^) generally kept track with culture density (Fig. 1**B**). In contrast, the Te-PC content per cell dry weight (mg g DW^−1^) increased rapidly over the first 8 days, and then plateaued for the remainder of the cultivation period. This observation could be explained by the light sensitive PC operon promoter, which shows increased activity in low light conditions. As the cell density increased from OD_750_ = 0.13 ± 0.03 at day 0 to 1.1 ± 0.1 at day 9, the light penetration through the carboys decreased by 7-fold (Lea-Smith et al., 2014). After day 10, the Te-PC content stayed relatively stable regardless of the presence or absence of antibiotic. However, by the end of the cultivation period (day 18), the Te-PC concentration and content of cultures without kanamycin was 11% and 18% higher, respectively, than those inoculated with kanamycin (*p* < 0.05). On day 18, the presence of the CT.353 TeBACD vector was confirmed in all carboys by PCR (see supplementary materials), which demonstrated that the cultures retained the self-replicating vector for producing Te-PC in the absence of a selective pressure for the duration of the cultivation period. This is consistent with recent work that has demonstrated that self-replicating RSF1010-based plasmids appear stable in cultures without antibiotic selection for at least six weeks (Nagy et al., 2021). Furthermore, the results suggested that the absence of kanamycin reduced cell stress and/or metabolic burden, which allowed for a small, but significant increase in Te-PC productivity.

In the second experiment, the culture growth conditions were modified to reach higher culture densities and further increase biomass accumulation. Previous work has shown that 2xBG11 medium can extend the growth phase of *Synechocystis* in saturating light (van Alphen et al., 2018). Thus, TeBACD cultures were grown in 2xBG11 medium and the intensity of white light was increased to the maximum light levels available (300 μmol photons m^−2^ s^−1^). Under these conditions, culture cell densities surpassing OD_750_ = 2 at 11 days compared to 15 days in the first experiment (Fig. 1**A**), and continued to increase for a longer period, with a maximum OD_750_ = 3.8 ± 0.2 reached after 24 days.

Culture growth was also examined under incrementally increased red light, from 160 to 640 μmol photons m^−2^ s^−1^ during the cultivation period, to try to postpone the onset of the stationary stage (Schuurmans et al., 2017). Regardless of the relative increase in overall light intensity, red light-grown cultures plateaued after 18 days, with a maximum OD_750_ = 2.2 ± 0.1 reached after 24 days (42% lower than white light-grown cultures). The lower growth rate could have been due to the reduced penetration of red light through the culture and liquid medium (Lea-Smith et al., 2014). Regardless of the differences in growth rates, the Te-PC content remained similar in white and red light conditions throughout the cultivation period (*p <* 0.05) (Fig. 1). Bland and Angenend (2016) also observed similar PC contents when *Synechocystis* was grown in equivalent levels of red or white light. However, red light-grown cultures were relatively paler by day 20. Measurements of Chl content on day 20 revealed a 35% lower Chl content in cultures grown in red light (3.2 ± 0.1 nmol Chl OD_750_^-1^) compared to those grown in white light (4.9 ± 0.0 nmol Chl OD_750_^-1^). The observed differences in Chl content likely reflect the ability of *Synechocystis* to adapt to the prevailing light conditions (Cordara et al., 2018).

The mean overall Te-PC content for the four independent growth experiments was 75.3 ± 1.7 mg g DW^−1^ (Fig. 1). Although the latter yields were lower (i.e. 33% reduced) compared to those from lab scale experiments (Table 1), the differences are likely attributed to more favourable growth conditions (i.e. light availability, aeration and mixing) at smaller scales. Nevertheless, the PC yields in the present study were comparable to those for *A. platensis* cultivated under non-optimised PBR conditions (Schipper et al., 2020), and are significantly higher than reports for thermophilic cyanobacteria, such as *T. elongatus* BP-1 (8.4), *T. vulcanus* (8.8) and *S. lividus* PCC 6715 (31) (Klepacz-Smolka et al., 2020, Liang et al., 2018). Although, the highest biomass productivity observed (i.e. 29.9 mg DW L^-1^ d^-1^ in white light and 2xBG11, Fig. 1**C** and Table 1) was at a lower end of that typically reported for *A. platensis* (23–750 mg DW L^-1^ d^-1^) (Schipper et al., 2020), Lindblad et al. (2019) have shown that *Synechocystis* can achieve a productivity of 185 mg DW L^-1^ d^-1^ in a scalable (1350 L) flat-panel PBR design under daylight conditions. Based on the Te-PC content in this study, the estimated Te-PC productivity of the TeBACD strain in an optimised flat-panel PBR (i.e. 13.9 mg PC L^-1^ d^-1^) could match that of *A. platensis* grown in open raceway ponds (6–15 mg PC L^-1^ d^-1^) (Uebel et al., 2019). Further optimisations of the growth medium may also provide additional increases in PC productivity (Zuorro et al., 2021).

**Table 1:** Comparison of parameters and results between lab scale (Puzorjov et al., 2021) and pilot scale production of Te-PC (this study).

**Parameter**	**Lab scale**	**Scale-up**
Culture volume	50 mL	20 L
Cultivation period (days)	5	24
Light conditions (μmol photons m-^2^ s^−1^)	50	300
Temperature (° C)	30 ± 1	30 ± 1
Aeration	Air	2% CO_2_ (v/v), 3 L min^−1^ flow rate
Biomass productivity (mg DW L^-1^ d^-1^)	173	29.9
Te-PC content (mg g^−1^ DW)	111.7 ± 1	75.3 ± 1.7
Te-PC productivity (mg L^-1^ d^-1^)	19.4	2.26
Cell harvesting approach	Centrifugation	Chitosan flocculation and centrifugation
Cell homogenisation approach	Bead-beating	High pressure homogenisation
Crude extract Te-PC purity (A_615_/A_280_)	0.83	1.25
Crude extract APC/PC ratio (A_652_/A_615_)	0.44	0.47
Crude extract Chl contamination (A_680_/A_615_)	0.08	0.17
Te-PC purification approach	Heat-treatment	Two-step ammonium sulfate with heat-treatment
Purified Te-PC purity (A_615_/A_280_)	0.71	2.9 ± 0.7
Purified Te-PC APC/PC ratio (A_652_/A_615_)	0.16	0.16
Purified Te-PC Chl contamination (A_680_/A_615_)	0.03	0.02
Te-PC recovery (%)	87 ± 2%	84 ± 12%

### 3.2 Culture harvest by chitosan-based flocculation

**Fig. 2:**
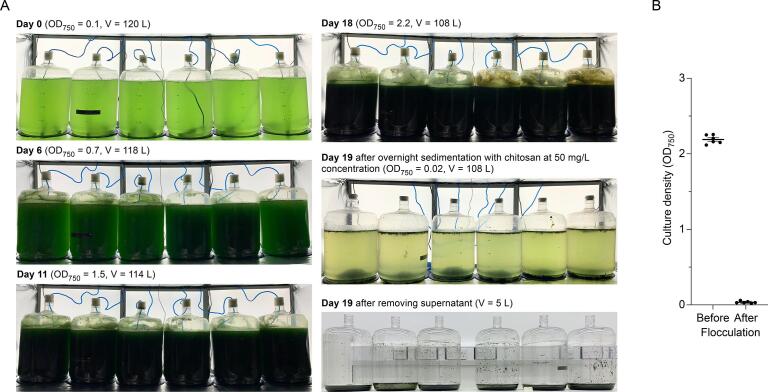
Example images of a pilot scale cultivation and flocculation of TeBACD cultures expressing thermostable phycocyanin. (A) Shown are six carboys (20 L) from Fig. 1**A** (three on the left) and **1B** (three on the right). The total culture volume decreased by 12 L over 18 days due to sample measurements and evaporation (i.e. 2 L per carboy). On day 18, chitosan was added (final concentration 50 mg L^-1^) and the cultures were left to sediment for 16 h at room temperature. On day 19 the bulk of the supernatant was removed, and the sedimented culture was centrifuged to obtain wet biomass. (B) Optical density (OD_750_) of the cultures before flocculation (day 18) and after flocculation (day 19). The error bars show the mean ± SE of culture densities in the six separate carboys.

The culture biomass was harvested by flocculation using chitosan, a naturally occurring polymer that is biodegradable and safe for human consumption. Chitosan is a positively charged linear polysaccharide that interacts with the negatively charged cyanobacterial cell wall to form bridges and facilitate flocculation. For the first experiment, addition of chitosan (50 mg L^-1^) to the 1xBG11 culture medium, which was at pH 7.2 at the end of the cultivation period, resulted in a near complete sedimentation of culture biomass (Fig. 2**A**). Following a 16-hour incubation, culture densities were decreased by 99 ± 1% (i.e. from 2.19 ± 0.02 to 0.03 ± 0.01) (Fig. 2**B)**. In the second experiment, the 2xBG11 medium was slightly more alkaline (i.e. pH 7.5) at the end of the cultivation period. Addition of chitosan to a sub-sample of the 2xBG11 culture medium at pH 7.5 reduced the culture density only by 75% (i.e. from 2.09 to 0.52), indicating that pH and/or the growth medium composition was suboptimal for efficient flocculation. As chitosan is most effective in a pH range from 6.0 to 7.2 (Divakaran and Pillai, 2002, Rojsitthisak et al., 2017), 10% CO_2_ was bubbled though the culture medium to temporarily reduce the pH to 6.8. Subsequent addition of chitosan resulted in a 95 ± 1% reduction in culture density.

Overall, treatment with chitosan was sufficient to sediment 97 ± 2% of the culture biomass across both experiments with initial culture densities (OD_750_) ranging between 1.9 and 4.1. The observed flocculation efficiencies were slightly better than those previously reported for *Synechocystis* (>90%) using 15 or 20 mg L^-1^ of chitosan at pH 7.0–7.2 (Divakaran and Pillai, 2002, Rojsitthisak et al., 2017). To the best of our knowledge, this is the first study to show that temporary culture pH adjustment using CO_2_ is sufficient to significantly increase flocculation efficiency. Considering that alkaline conditions are common for cultivation of most algae and cyanobacteria, the approach described here could further simplify the use of chitosan either as an efficient microalgal biomass flocculant or as a PC purifying agent (Fekrat et al., 2019, Sukhinov et al., 2021).

### 3.3 Extraction of phycocyanin from *Synechocystis* cells using high pressure homogenisation

High pressure homogenisation (HPH) was used to disrupt the wet TeBACD biomass collected from the second experiment and generate two crude Te-PC extracts (i.e. for red- and white light-grown cultures). Previous studies have demonstrated that HPH can be an effective, time-efficient and scalable technique for crude mechanical disruption of algal (e.g. *Nannochloris oculata* UTEX 1998) and cyanobacterial cells (e.g. *Arthrospira platensis*) (Ruiz-Domínguez et al., 2019; Samarasinghe et al., 2012). Furthermore, cell disruption using HPH is an energy efficient method for mechanical extraction (i.e. in terms of kWh kg^−1^ DW biomass) and does not require the addition of chemicals as for non-mechanical extraction methods (e.g. solvents or enzymes) (Safi et al., 2017). However, the efficiency of cell disruption by HPH is highly dependent on the conditions used (i.e. the pressure applied and number of passes) and the thickness and chemical composition of the cell wall in different species (Papachristou et al., 2020). To the best of our knowledge, disruption of *Synechocystis* cells using HPH has not been reported previously.

**Fig. 3:**
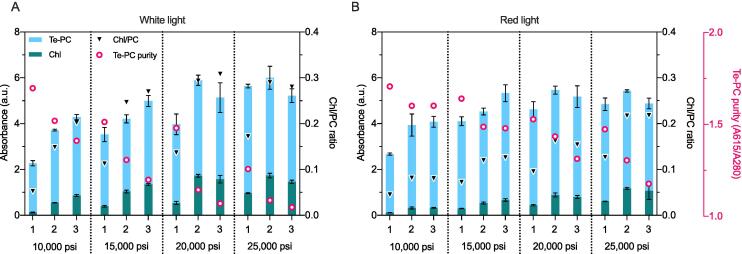
Characterisation of disrupted *Synechocystis* wet biomass. Cultures grown in white light (300 μmol photons m^−2^ s^−1^) (A) or red light (B) were disrupted using a high-pressure homogeniser (HPH). The number of HPH passes (1–3) and the pressure used (10,000–25,000 psi) are noted on the x-axis. Crude estimates of phycocyanin (PC), chlorophyll *a* (Chl) and total protein were measured after each pass at 615 nm, 680 nm and 280 nm, respectively. The bars are superimposed, and the error bars show the mean ± SD of three technical replicates. PC purity and Chl contamination was measured as the ratio of PC to total protein and Chl to PC absorption peaks, respectively. Prior to homogenisation, the biomass was diluted to 12.4–13.9 g DW L^-1^. (For interpretation of the references to colour in this figure legend, the reader is referred to the web version of this article.)

The *Synechocystis* cell wall is approximately 60 µm thick and consists of a plasma membrane, a peptidoglycan layer, outer membrane, and S-layer (Trautner & Vermaas, 2013). The peptidoglycan layer in *Synechocystis* sp. is considerably thicker (∼30 nm) and contains a higher degree of cross-linking between peptidoglycan chains (56–63%) compared to most gram-negative bacteria (2–6 nm; 20–33%), which makes *Synechocystis* sp. more resistant to mechanical stress. Although *Synechocystis* cells can be disrupted by a variety of methods, excessive cellular disruption may result in release of undesirable contaminants. For example, sonication of *Synechocystis* cells has previously resulted in extraction of both soluble and membrane-bound proteins, leading to high levels of Chl contamination in the crude PC extract (Zavrel et al., 2018). Here, the efficiency of cell disruption was investigated following 1–3 passes in HPH at 10,000–25,000 psi using absorption spectroscopy (Fig. 3). Prior to cell disruption, the biomass was washed with alkaline PBS (pH 8.8) to remove residual chitosan.

Consistent with earlier culture measurements, crude Te-PC extracts from cultures grown in red light showed a reduction in Chl contamination for the majority of HPH conditions tested (Fig. 3). Te-PC content in the supernatant remained similar following one, two or three passes at 25,000 psi regardless of the differences in Chl content between cultures grown in red or white light (Fig. 3). Notably, one pass at 25,000 psi resulted in the highest Te-PC purity and lowest Chl contamination achievable while maximising Te-PC extraction for both red and white light cultures, suggesting that one pass at 25,000 psi was sufficient to maximise the extraction of Te-PC from *Synechocystis* biomass. However, the Chl contamination levels were still significantly higher than those observed using bead-beating approach at a lab scale (Table 1). Although a previous a report has indicated that these pressures can reduce PC yields from *A. platensis* (Ruiz-Domínguez et al., 2019), no evidence of Te-PC degradation was observed after additional passes regardless of the pressure used.

### 3.4 Purification of thermotolerant phycocyanin using ammonium sulphate and heat treatment

**Fig. 4:**
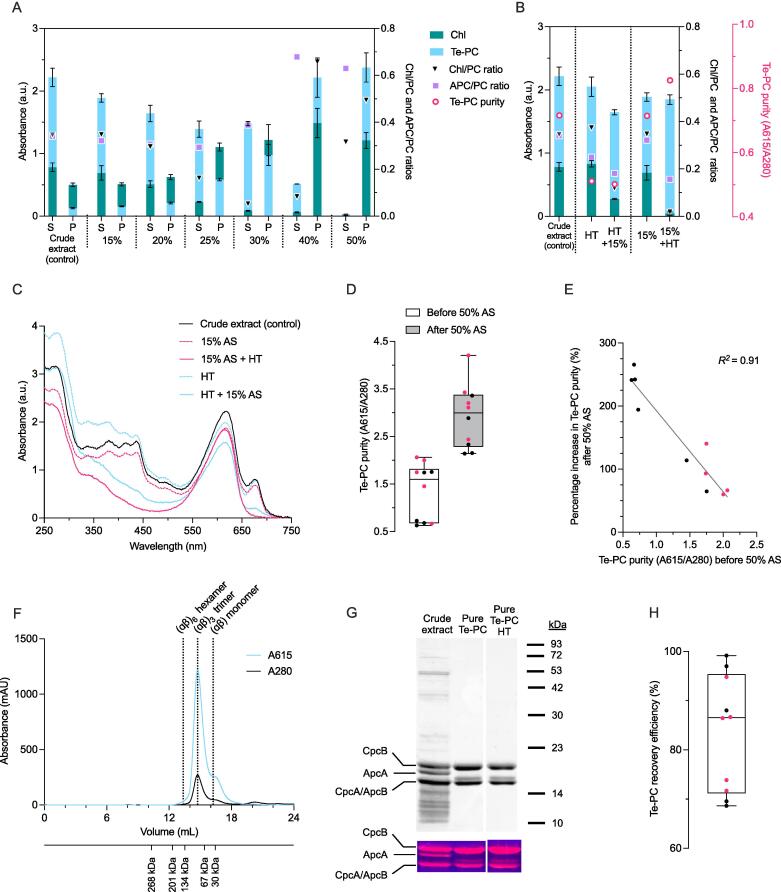
Purification of Te-PC using a two-step ammonium sulphate precipitation and heat treatment. (A) The effect of increasing ammonium sulphate (AS) on precipitation of Te-PC and Chl from the supernatant (S) into the pellet (P). (B) The effect of heat treatment (HT, 60 °C for 15 min) applied before or after the addition of 15% AS. The bars for A and B are superimposed, and the error bars show the mean ± SD of three technical replicates. Estimates of Te-PC, allophycocyanin (APC), Chl and total protein was measured at 615 nm, 652 nm, 680 nm and 280 nm, respectively. (C) Absorption spectra of Te-PC supernatants at each purification step shown in (B). (D) Box and whisker plot comparing Te-PC purity after a first step 15% AS + HT step purification (i.e before 50% AS) and then after a second step, where the resuspended pellet was precipitated in 50% AS. Results are shown for 10 independent experiments using Te-PC crude extract obtained from red light (red filled dots) and white light (black filled dots) from both experiments. (E) Correlation between the Te-PC purity of the sample before the second 50% AS precipitation step and the percentage improvement following the 50% AS precipitation step (R^2^ = 0.91, n = 10, *p* < 0.05). (F) Size-exclusion chromatography profile of purified Te-PC after dialysis and freeze drying. The elution volumes of the molecular weight standards are shown under the *x*-axis. (G) SDS-PAGE of the crude PC extract, purified Te-PC and purified Te-PC following heat-treatment at 70 °C for 180 min. Peptides were visualized with Coomassie brilliant blue staining. Zinc-induced fluorescence of chromophorylated phycobiliproteins under UV light is shown below. Abbreviations: ApcA/ApcB, allophycocyanin ⍺- and β-subunits; CpcA/CpcB, phycocyanin ⍺- and β-subunits; (H) Box and whisker plot showing the Te-PC recovery efficiency in the resuspended pellet following 50% AS precipitation compared to the initial crude Te-PC extract. (For interpretation of the references to colour in this figure legend, the reader is referred to the web version of this article.)

Following cell disruption, a two-step ammonium sulfate (AS) precipitation approach was employed to test if Te-PC could be purified from the crude Te-PC extract (Mogany et al., 2019, Silva et al., 2009). The concentration of AS used in the first step was initially optimised to reduce the loss of Te-PC and maximise APC and Chl removal from the supernatant (Fig. 4**A**). The relative concentrations of Te-PC, APC and Chl were estimated in both the supernatant and pellet for increasing concentrations of AS and compared to the control (i.e. the crude Te-PC extract).

The concentration of Te-PC in the supernatant decreased gradually from 15 to 30% AS. At 40% AS, Te-PC was 77% lower than the control. In contrast, the APC/PC ratio at 40% AS increased from 0.34 to 0.68, suggesting that APC is precipitated at a higher AS saturation compared to Te-PC. Complete precipitation of Te-PC and APC was achieved at 50% AS. Chl levels remained similar in the supernatant from 15 to 20% AS, but then dropped sharply at 25–30% AS. The Chl/PC ratio in the supernatant was reduced from 0.35 in the control to the lowest value measured of 0.05 at 30% AS. However, this was accompanied by a 43% reduction in Te-PC yields. Therefore, removal of Chl was not achievable without a significant reduction in PC yield in the first step of AS precipitation.

A heat-treatment (HT) was then tested to reduce the levels of Chl and further increase the purity of Te-PC in the supernatant. A HT of 60 °C for 15 min was applied to the crude Te-PC extract either before or after the addition of 15% AS and compared to the control (i.e. the crude Te-PC extract without HT) (Fig. 4**B and 4C**). HT of the crude extract reduced the APC/PC ratio by 26% from 0.34 to 0.25 but had a negative impact on the Te-PC purity, which decreased by 29% from 0.72 to 0.51. HT had no significant effect on the Chl/PC ratio of the sample. Subsequent addition of 15% AS after HT further reduced the APC/PC ratio to 0.18 and the Chl/PC ratio by 67% from 0.37 to 0.12. However, the Te-PC content in the supernatant also decreased by 27% (*p* < 0.05).

Addition of 15% AS alone had little impact on the composition of the crude Te-PC extract (Fig. 4**B and 4C**). However, when 15% AS was followed by HT, the APC/PC and Chl/PC ratios decreased by 54% (0.16) and 94% (0.02), respectively. The latter APC/PC ratio matched previous lab scale results following heat treatment (Table 1) (Puzorjov et al., 2021). A slight reduction in estimated Te-PC content (*p* < 0.05) could be attributed to near complete removal of Chl and APC, both of which contribute to the Te-PC peak at 615 nm (Puzorjov et al., 2021, Zavrel et al., 2018). Therefore, both APC and Chl contaminants could be efficiently removed from the crude Te-PC extract using a combination of 15% AS precipitation followed by HT.

The second precipitation step at 50% AS was performed on the supernatants of 15% AS + HT samples obtained from 10 separate Te-PC purification experiments from both experiments to further separate Te-PC from soluble contaminants and improve the PC purity (Mogany et al., 2019, Silva et al., 2009). Precipitation at 50% AS demonstrated that the two-step AS approach led to a more than a two-fold increase in PC purity from 1.3 ± 0.6 to 2.9 ± 0.7 (*p* < 0.05) (Fig. 4**D**). The percentage increase in Te-PC purity was inversely correlated with the Te-PC purity before precipitation (*R^2^* = 0.91, *n* = 10, *p* < 0.05) (Fig. 4**E**). However, the highest Te-PC purity values (>3.0) were only achieved in samples with an initial PC purity of 1.5 or above. Thus, maximising the purity of PC in previous steps was important to achieve high PC purities downstream. In particular, the HPH extraction could be further optimised to reduce the release of contaminants from ruptured cells (Fig. 3). Additionally, the crude PC extract could be purified with chitosan and activated charcoal prior to the first step AS precipitation (Sukhinov et al., 2021).

The purified Te-PC sample was further analysed by size-exclusion chromatography (SEC) and SDS-PAGE. The elution profile at 615 nm showed a small leading peak at 13.4 mL, a major peak at 14.7 mL and a trailing shoulder at 16.3 mL, which represented approximately 5%, 80% and 15% of the total sample, respectively (Fig. 4**F**). Based on correlation of Te-PC elution volumes with molecular weight standards, and determination of apparent molecular weights, these peaks corresponded to (ɑβ)_6_ hexamers (∼200 kDa), (ɑβ)_3_ trimers (∼95–100 kDa) and (ɑβ) monomers (∼30–32 kDa) of Te-PC, which is consistent with data previously reported for PC (Song et al., 2013). The only other portion of the elution profile that showed a signal at 280 nm was eluted at 20–21 mL. However, the apparent molecular size of components in this fraction was ∼ 1 kDa or less, and could constitute a range of small molecules outside of the dynamic range of the column, including sample buffer molecules, metabolites or small peptides. Protein separation by SDS-PAGE also clearly demonstrated a significant reduction in background protein content and the absence of the ⍺-subunit of allophycocyanin (ApcA) in purified Te-PC samples compared to the crude extract (Fig. 4**G**). The β-subunit of allophycocyanin (ApcB) is similarly sized to the ⍺-subunit of phycocyanin (CpcA), thus it is difficult to clarify if the former was reduced. However, previous work in *Synechocystis* has shown that ApcA is less thermostable that ApcB (Chen et al., 2016), so it was reasonable to speculate that ApcB was also removed during purification.

The purified Te-PC sample also had an additional faint band above CpcA that was not present in the crude extract (Fig. 4**G**). The new band showed zinc-induced fluorescence under UV-light, which indicated the presence of phycobilin chromophore. Additional heat treatment of purified Te-PC for 180 min at 70 °C had no impact on the sample, which suggested that the new band was not a Te-PC breakdown product produced during the heat-treatment purification step. Previous work has shown that the β-subunit of phycocyanin (CpcB) has a lower molecular weight when partially chromophorylated (Shen et al., 2008). Therefore, the new band in the pure Te-PC sample could be a partially chromophorylated CpcB subunit that was enriched during the purification process. Overall, the SEC and SDS-PAGE data showed that the majority of protein contaminants, including APC, were removed during two-step AS precipitation combined with HT.

The PC recovery efficiency following two-step AS precipitation was measured as the difference between the PC absorption peak at 615 nm in the crude PC extract and after resuspending the pellet following precipitation at 50% AS (Fig. 4**H**). The mean PC recovery efficiency from the 10 precipitation experiments was 84 ± 12%. The PC recovery results were similar to those previously reported using optimisation of the two-step AS purification protocol. For example, 82% PC recovery was achieved from *G. sulphuraria* following 25% and 50% AS precipitation steps (Moon et al., 2014). For PC from *A. platensis*, Silva et al. (2009) reported 84% PC recovery following 20% and 50% AS precipitation steps. In the present study, crude PC extracts with purity values below 0.8 showed a 250 ± 14% increase in Te-PC purity from 0.66 ± 0.03 to 2.31 ± 0.14 compared to a 70% increase from 0.52 to 0.88 reported by Silva et al. (2009).

Separation of PC from APC is typically achieved using a combination of gel filtration chromatography and ion exchange chromatography (Shen et al., 2008). However, this approach can be disadvantageous due to the numerous of steps involved in the purification process, which can result in challenges in scale-up, increased costs and downstream processing times, and a significant loss of product yield (Phong et al., 2018, Porav et al., 2020, Scorza et al., 2021). More recently, aqueous two-phase systems (ATPS, also known as liquid biphasic systems) in combination with ultrafiltration have shown promising phycobiliprotein purification and separation results, with high PC recoveries (>80%) and PC purities (>3.4) achieved for *A. platensis* AICB49 and *Synechocystis* sp. AICB51 (Porav et al., 2020). However, efficient ATPS separation requires the use of polymers, such as polyethylene glycol and dextran, which are difficult to recover and can be expensive (Varadavenkatesan et al., 2021). Furthermore, the recovery efficiency and purity of phycobiliproteins obtained using ATPS is dependent on several crucial parameters (e.g. liquid phase components, sample concentrations and volume ratios, crude extract quality, pH and temperature), which can require extensive and time-consuming optimisation (Phong et al., 2018, Varadavenkatesan et al., 2021). In contrast, the purification approach presented here for Te-PC requires protein salting-out using AS and simple HT, which are straightforward to perform, inexpensive and scalable, yet provide PC recovery and PC purity similar to the best results obtained using ATPS.

A cost analysis of the production and purification process presented here indicated that the production cost of one gram of purified Te-PC (A_620_/A_280_ > 3.0) was £1,230 (see supplementary materials) (Tredici et al., 2016). At present, the equivalent cost of one gram of analytical grade PC (A_620_/A_280_ > 3.5, A_651_/A_620_ < 0.3) purified from *A. platensis* is £102,600 (52468-5MG-F, Sigma-Aldrich). Therefore, the scale-up production and purification of a high-value thermostable Te-PC presented here is economically viable for industrial production.

### 3.5 Analysis of PC stability at different temperature and pH conditions

**Fig. 5:**
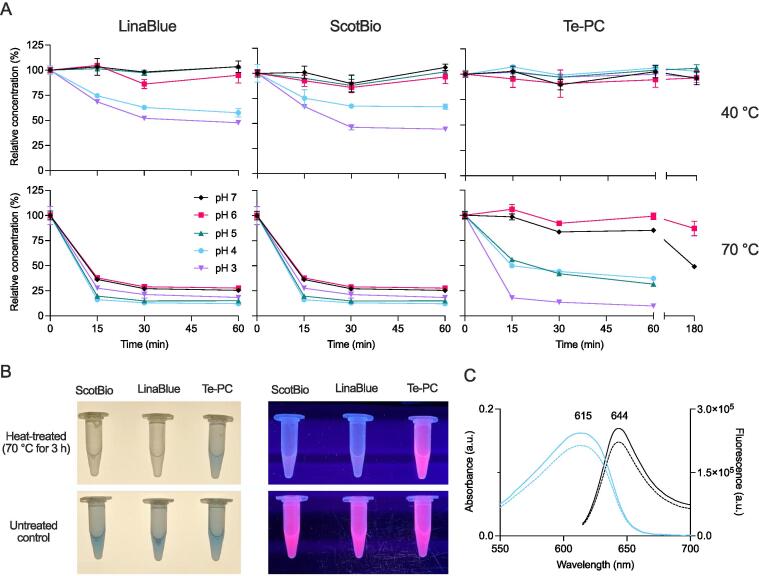
Comparison of stability between Te-PC and PC from *A. platensis*. (A) Samples were incubated for 15, 30 and 60 min at 40 °C and 70 °C in solutions buffered to pH values from 3 to 7. Te-PC samples at 40 °C and 70 °C (pH 6–7) were incubated for 180 min. Sample stability is expressed as a relative percentage of the PC content of untreated controls (all at 0.1 g L^-1^). Values are the mean ± SD of three technical replicates. LinaBlue and ScotBio are commercially available PC samples extracted from *A. platensis*. (B) Examples of PC samples heat-treated at 70 °C for 180 min at pH 6 (top) and untreated controls (bottom) in ambient light (left) and UV light (312 nm, right). (C) Absorption (blue) and fluorescence emission spectra (black, excited at 590 nm) of untreated controls (solid lines) and heat-treated Te-PC (dashed lines) from (B). (For interpretation of the references to colour in this figure legend, the reader is referred to the web version of this article.)

The thermostability of purified Te-PC following two-step AS precipitation was compared against two commercially available PC samples extracted from *A. platensis* (LinaBlue, DIC; ScotBio). The samples were evaluated at 40 °C and 70 °C, and pH values from 3 to 7, over a 60 or 180 min incubation period (Fig. 5**A**). All three samples were stable at 40 °C between pH 5 to 7. However, both PC samples from *A. platensis* showed decreased stability below pH 5, while Te-PC showed no signs of degradation. The results here also indicated that Te-PC is more stable at pH 3 and 4 than PC extracted from the thermoacidophilic red microalga *Cyanidioschyzon merolae*, which degraded to 37% and 50% of the initial values, respectively, after incubation at 27 °C for 180 min (Rahman et al., 2017). The known mechanisms associated with the increased stability of PC from extremophiles has been recently reviewed (Puzorjov & McCormick, 2020). At 70 °C, both PC samples from *A. platensis* were rapidly degraded after 15 min regardless of the pH. In contrast, Te-PC appeared stable for the duration of the assay at pH 6 (*P* > 0.05) and decreased by 15% and 51% (*p* < 0.05) at pH 7 after incubation 60 and 180 min, respectively. The stability of Te-PC decreased below pH 6 and showed similar rates of degradation to PC at pH 3. Overall, Te-PC obtained from *Synechocystis* in this pilot study performed significantly better than two commercial PC products at 40 °C in pH 3 to 7 and at 70 °C in pH 6 and 7. In contrast to the two PC samples, Te-PC retained its visible blue colour and red fluorescence under UV light after incubation at 70 °C for 180 min at pH 6 (Fig. 5**B**). Quantitative analyses of the Te-PC samples showed a slight reduction in absorbance and fluorescence emission intensity (12% for both) compared to the untreated control, but no change in the positions of the absorbance and fluorescence peaks at 615 nm and 644 nm, respectively (Fig. 5**C**). These results were in agreement with the degradation temperature of Te-PC and of the native PC extracted from *T. elongatus* reported previously using fluorescence spectroscopy (Puzorjov et al., 2021).

## 4 Conclusion

In this study, a scalable production, harvesting and purification process for producing a thermostable PC from *Synechocystis* was demonstrated. Production of Te-PC from a self-replicating vector was unaffected by the absence of antibiotic selection. Efficient biomass harvesting using chitosan could be optimised by CO_2_ bubbling to adjust culture pH. A single pass at 25,000 psi using HPH maximised Te-PC extraction and reduced potential Chl contamination in the crude extract. Further purification of Te-PC using a heat treatment and two-step AS precipitation removed contaminants and improved Te-PC purity values to near analytical grade levels.

### CRediT authorship contribution statement

**Anton Puzorjov:** Conceptualization, Writing – original draft, Investigation, Methodology, Formal analysis, Visualization, Project administration. **Suleyman Mert Unal:** Investigation. **Martin A. Wear:** Methodology, Investigation. **Alistair J. McCormick:** Supervision, Writing – original draft, Funding acquisition, Resources.

## Declaration of Competing Interest

The authors declare that they have no known competing financial interests or personal relationships that could have appeared to influence the work reported in this paper.
