# Supplementary material for: Pilot scale production, extraction and purification of a thermostable phycocyanin from Synechocystis sp. PCC 6803
Source: Bioresour Technol. 2022 Feb;345:126459. doi: 10.1016/j.biortech.2021.126459 (PMC8811538; doi:10.1016/j.biortech.2021.126459)
Supplement: Supplementary data 1 [file mmc1.zip › mmc1.docx]

**Pilot scale production, extraction and purification of a thermostable phycocyanin from *Synechocystis* sp. PCC 6803**

 Anton Puzorjov^1^, Suleyman Mert Unal^1^, Martin A. Wear^2^, Alistair J. McCormick^1,†^

^1^SynthSys & Institute of Molecular Plant Sciences, School of Biological Sciences, University of Edinburgh, Edinburgh, EH9 3BF, UK

^2^The Edinburgh Protein Purification Facility, University of Edinburgh, Edinburgh EH9 3JR, UK

**Supplementary materials**


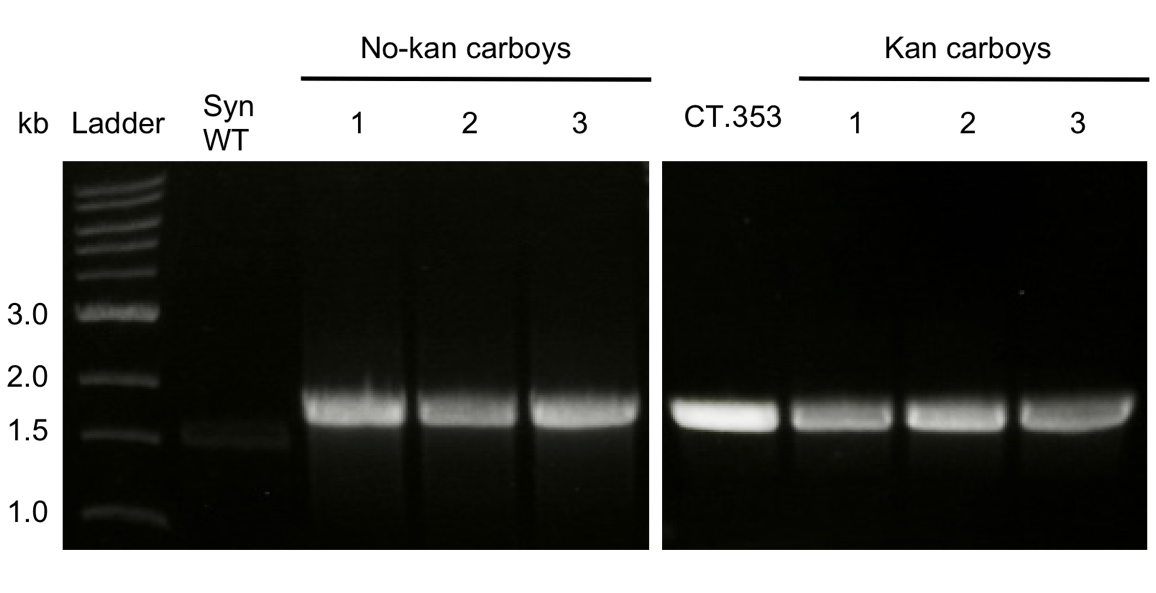


**Figure S1.** The self-replicating CT.353 TeBACD vector was retained in the strain regardless of antibiotic selection. Gel electrophoresis showing amplicons of a portion of the TeBACD operon and the pPMQAK1-T backbone (1818 bp) amplified from DNA extracted from culture samples harvested on day 18 of experiment 1. A wild-type *Synechocystis* sp. PCC 6803 strain (Syn WT) and a purified CT.353 vector were used as a negative and positive controls, respectively. Primers used for amplification: 5’ TAGCTGAGGGCGTTAATGGCG 3’ and ‘5 TTGAGTGAGCTGATACCGCT 3’. Both are included in the supplementary material file “CT.353 TeBACD.gb” as Primer 1 and Primer 2, respectively.

**Table S1.** Operational and capital cost analysis of production and purification of 1 gram of pure Te-PC (A615/A280 > 3.0). Estimates are based on 2020 costs in the UK. Labour is based on that for UK grade 6 research staff, electricity is based on the average cost per kWh in the UK (£0.172 kWh^-1^), and rent is based on standard estate charges at the University of Edinburgh. Indirect operating and capital costs were estimated as described in Tredici et al. (2016). Due to the relatively low capital requirement, capital was assumed to be readily available and no borrowing was necessary. The annual capital expenditure was calculated by dividing the total capital expenditure by the lifespan of the asset (10 years) using a straight-line depreciation method. Calculations are based on fourteen 120 L batch cultures (grown under 300 μmol photons m^-2^ s^-1^ of white light in 2xBG11 medium) per year and a cultivation period of 24 days. Abbreviations: CAPEX: capital expenditure; OPEX: operating expenditure; TDC: total direct capital costs; TDO: total direct operating costs.

|  |  | **Annual cost (£)** |  |
| --- | --- | --- | --- |
| **DIRECT OPERATING COSTS** | |  |  |
|  | Labour (one full-time employee) | 36,500 |  |
|  | Chemicals | 4,311 |  |
|  | Electricity | 591 |  |
|  | Rent | 21,900 |  |
|  | **Total direct operating costs (TDO)** | 63,302 |  |
| **INDIRECT OPERATING COSTS** | |  |  |
|  | Maintenance (5% of TDC) | 3,165 |  |
|  | Overhead (10% of TDO) | 6,330 |  |
|  | Administration (10% of TDO) | 6,330 |  |
|  | **Total indirect operating costs** | 15,826 |  |
| **TOTAL OPEX** | | 79,128 |  |
|  |  | **Cost (£)** | **CAPEX per annum (£)** |
| **DIRECT CAPITAL COSTS** | |  |  |
|  | PBR carboys (25 L) | 120 | 12 |
|  | Piping, ﬁttings and valves | 900 | 90 |
|  | Machinery and equipment | 85,300 | 8,530 |
|  | Electrical equipment, instrumentation and controls | 620 | 62 |
|  | **Total direct capital costs (TDC)** | 86,940 | 8,694 |
| **INDIRECT CAPITAL COSTS** | |  |  |
|  | Engineering & supervision (5% of TDC) | 4,347 | 435 |
|  | Installation (10% of TDC) | 8,694 | 869 |
|  | Taxes & insurance (1% of TDC) | 869 | 87 |
|  | **Total indirect capital costs** | 13,910 | 1,391 |
| **TOTAL CAPEX** | | 100,850 | 10,085 |
|  |  |  |  |
|  |  |  |  |
|  | Total annual cost (OPEX and CAPEX) | **£ 89,212.66** |  |
|  | Total pure Te-PC yield (g) per year from 14 batches | **72.0** |  |
|  | Production cost of 1 gram of pure Te-PC | **£ 1,239** |  |
